# Intelligent Nanoparticles With pH-Sensitive Co-Delivery of Temozolomide and siEGFR to Ameliorate Glioma Therapy

**DOI:** 10.3389/fgene.2022.921051

**Published:** 2022-07-12

**Authors:** Zhen Wang, Yuyang Liu, Yong Xiao, Yandong Xie, Ran Wang, Yiding Zhang, Qi Zhou, Liang Liu, Shuo Sun, Hong Xiao, Yuanjie Zou, Kun Yang, Xiang Li, Mengjie Zhao, Yifang Hu, Hongyi Liu

**Affiliations:** ^1^ Department of Neurosurgery, The Affiliated Brain Hospital with Nanjing Medical University, Fourth Clinical College of Nanjing Medical University, Nanjing, China; ^2^ Department of Neuro-Psychiatric Institute, The Affiliated Brain Hospital with Nanjing Medical University, Nanjing, China; ^3^ Department of Neurosurgery, The Affiliated Hospital of Xuzhou Medical University, Xuzhou, China; ^4^ Department of Geriatric Endocrinology, The First Affiliated Hospital of Nanjing Medical University, Nanjing, China

**Keywords:** glioma, pH-responsive polymeric nanocarrier, synergistic therapy, TMZ, siEGFR

## Abstract

Glioblastoma (GBM) is one of the most lethal forms of human cancer, with very few long-term survivors. In addition to surgery, chemotherapy is still an important strategy. Unfortunately, GBM chemotherapy faces two main challenges: first, in GBM, epidermal growth factor receptor (EGFR) overexpression results in chemoresistance; second, temozolomide (TMZ) lacks target specificity, which can lead to a reduction in the concentration and side effects in GBM. Nowadays, with the development of nanomedicine systems for applications in tumor therapies, increasing anticancer efficacy and reducing side effects with multi-drug delivery are huge advantages. In this study, pH-sensitive and GBM-targeting nanovesicle (Tf-PEG-PAE(SS)) was fabricated. The chemotherapy drug (TMZ) and EGFR inhibitor (EGFR-siRNA) were co-encapsulated in the nanocarrier, and their anticancer outcomes were investigated in detail. *In vitro* experiments have shown that the nanocarrier transports TMZ and EGFR-siRNA efficiently into U87 cells, causing a vigorous apoptotic response by silencing the proliferative EGFR gene and increasing the drug concentration of TMZ simultaneously. An experimental study in mice bearing orthotropic glioma revealed that the accumulated nanocarriers in the tumor site could inhibit the tumor growth and prolong the mice survival remarkably through the intracranial injection of Tf-PEG-PAE(SS)/TMZ@siEGFR. The drug co-delivery system could extend the blood circulation time and offer a new strategy to treat glioblastoma.

## Introduction

Glioblastoma (GBM), a primary malignant brain tumor of grade 4, is one of the most common and aggressive primary brain tumors in adults (Wen and Kesari, 2008; Wu et al., 2022). Even though comprehensive treatment options include traditional surgery, radiation, and chemotherapy, the median survival is only 14.6 months, and the 5-year survival rate is <5% (Stupp et al., 2005). Presently, temozolomide (TMZ), as a standard chemotherapeutic treatment for GBM, has shown confirmed success in improving the survival rate of patients suffering from this disease (Ziu et al., 2015; Bi et al., 2018). However, TMZ’s clinical effectiveness is restricted by the following problems: 1) the acquired drug resistance of glioma cells to TMZ during chemotherapy (Jiapaer et al., 2018), 2) damage to normal cells is caused by the indiscriminate attack on DNA (Kim et al., 2015), and 3) due to the blood–brain barrier (BBB) preventing orally administered or intravenously administered TMZ from entering the brain; thus, the effectiveness of TMZ as an antiglioma treatment is greatly reduced (Yao et al., 2022). Although the BBB restriction and the indiscriminate attack on DNA could be resolved by nanoparticles (NPs) as vehicles to targeted delivery of TMZ (Cheng et al., 2021), drug resistance remains a huge challenge for TMZ to perform its best function (Lee, 2016). Therefore, there is an urgent need for a new strategy to conquer TMZ resistance and increase the therapeutic benefits.

According to clinical pathological findings, the expression of epidermal growth factor receptor (EGFR) in GBM tissues was clearly higher than that in normal brain tissues (An et al., 2018). Statistically, more than half of initial GBMs have gene changes of EGFR, a prominent oncogenic driver of chemoresistance (Eskilsson et al., 2018; Ciechomska et al., 2020). Through downstream effectors such as the Ras/Raf/MAPK and PI3K/Akt/mTOR signaling pathways, EGFR activation could induce tumor cell proliferation and survival (Fan and Weiss, 2012; Gao et al., 2013). EGFR also protects against DNA-damaging substances through a variety of mechanisms including enhanced DNA strand break repair, thus weakening the efficacy of TMZ (Vengoji et al., 2019). Therefore, downregulation of the EGFR expression could cause apoptosis and inhibit proliferation in glioma cells (Ghildiyal et al., 2013). At present, small-molecule inhibitors of EGFR have been proven to increase the sensitivity of temozolomide-resistant glioma (Vivanco et al., 2012). Sharifi et al. (2019) covered that a combination of TMZ and the EGFR inhibitor, ZR 2002, obviously improved the survival of mice harboring intracranial mesenchymal temozolomide-resistant glioma cell line. Chen et al. reported that LRIG1 could reverse multidrug resistance (MDR) by inhibiting EGFR in GBM (Liu et al., 2015). These citations prompted that a combinative strategy using TMZ and EGFR inhibitor may overcome temozolomide resistance and enhance tumor apoptosis. Since RNA interference (RNAi) has such a specific and robust effect on the gene expression, small interfering RNA (siRNA) has been found to be a valuable therapeutic agent for the gene expression control (Hu et al., 2019; Hu et al., 2020). Hence, silencing EGFR through EGFR-siRNA (siEGFR) is a promising therapeutic manner. However, siRNA therapy is generally characterized by weak targeting, poor cell membrane penetration, degradation by enzymes, and a poor sensitization effect, which limits the therapeutic effect (Gilleron et al., 2013; Nikam and Gore, 2018; Singh et al., 2018). Therefore, the development of a new delivery system that can simultaneously deliver TMZ and siEGFR has become the focus of research.

**FIGURE 1:**
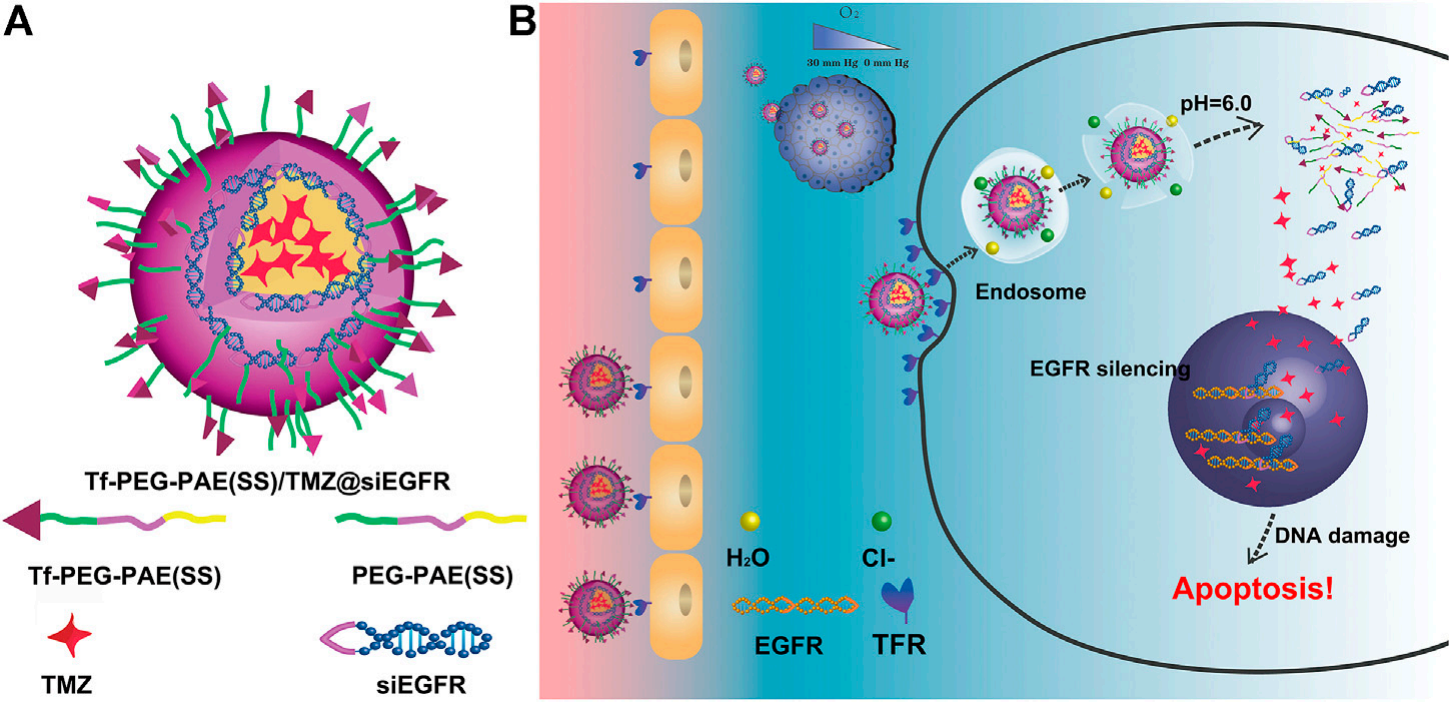
Schematic illustration of the Tf-PEG-PAE(SS)/TMZ@siEGFR micelle. **(A)** Main components of Tf-PEG-PAE(SS)/TMZ@siEGFR. **(B)** Tf-PEG-PAE(SS)/TMZ@siEGFR nanoparticles dramatically gather in glioma by Tf-mediated transcytosis strategy and then get rid of the endosome releasing siEGFR and TMZ; the siEGFR silences gene expression by reinforcing the anticancer effect of TMZ.

In this study, we developed a pH-responsive chemosensitizer-prodrug system Tf-PEG-PAE (SS) to co-deliver siEGFR and TMZ into glioma cells, with increasing TMZ sensitivity. Tf-PEG-PAE (SS) was self-assembled into micelles encasing TMZ in the hydrophobic core by the hydrophobic contact. The siEGFR was then electrostatically complexed with the cationic micelle. The micelle was expected to be quickly absorbed into glioma cells with the transferrin (Tf)-targeting molecule on the surface layer, where the transferrin receptor (TfR) is greatly overexpressed relative to the normal cells (Sun et al., 2020) (Figure 1). Tf-PEG-PAE(SS)/TMZ@siEGFR may benefit the penetration of siEGFR and TMZ across the blood–brain barrier (BBB) and protect siEGFR from degradation. Consequently, Tf-PEG-PAE(SS)/TMZ@siEGFR increased the sensitivity of glioma cells to TMZ, improving the standard-of-care therapy.

## Materials and Methods

### Materials

TMZ was purchased from Dalian Meilun Biotechnology Company (Dalian, China). Tf, PAE-PEG-NHS and Cy5-TMZ were obtained from Xian Ruixi Biotechnology Company (Xian, China). siEGFR-targeting EGFR mRNA (sense strand: 5′-CAAAGUGUGUAACGGAAUAdTdT-3′) and FAM-siEGFR were purchased from Gene Pharma Company Limited (Shanghai, China); 3-(4,5-dimethylthiazol- 2-yl)-2,5-diphenyltetrazolium bromide (MTT), 4′,6-diamidino-2-phenylindole dihydrochloride (DAPI), and AnnexinV-fluorescein isothiocyanate (FITC)/propidium iodide (PI) were purchased from Nanjing Key GEN Biosciences Company (Nanjing, China); 5-ethynyl-2-deoxyuridine (EdU) was purchased from Ribo Biotechnology Company (Guangzhou, China). Alanine aminotransferase (ALT), aspartate transaminase (AST), blood urea nitrogen (BUN), and Creatinine (CREA) assay kits were obtained from Jian Cheng Biotechnology Company (Nanjing, China).

The U87 glioma cell line was obtained from the National Collection of Authenticated Cell Cultures and cultured in a DMEM medium containing 10% fetal bovine serum (FBS) at 37°C, 5% CO_2_ atmosphere.

Female BALB/c nude mice (5 weeks old, 15–18 g) were purchased from Beijing Vital River Laboratory Animal Technology Company (Beijing, China). All animal experiments comply with the regulations of the experimental animal management committee of Nanjing Medical University.

### Synthesis of the Tf-PEG-PAE(SS) Polymer

First, 100 mg of 2,2 ′- disulfide diethanol, 2.4 eq. of acryloyl chloride, and 2.4 eq. of triethylamine were dissolved in 5 ml of anhydrous dichloromethane and stirred at 25°C for 24 h. The reaction solution was precipitated into water three times, dried with anhydrous sodium sulfate, and then poured it into a large amount of glacial ether for precipitation. The mixture was centrifuged to collect the product and then vacuum-dried to obtain 2,2' -disulfide diethanol diacrylate. Second, 1 g of acrylate-PEG5k-COOH, 10.0 eq. of 2,2 ′- disulfide diethanol diacrylate, and 11 eq. of 1,3-bis (4-piperidine) propane were dissolved in 10 ml of chloroform and stirred for 48 h at 55°C. The reaction solution was concentrated and poured into a large amount of ice ether for sedimentation. The mixture was centrifuged and vacuum-dried to obtain PAE(SS)-PEG5k-COOH. Third, 1 g of PAE(SS)-PEG5k-COOH, 3.0 eq. of EDC, and 3.0 eq. of N-hydroxysuccinimide were dissolved in 10 ml of chloroform and stirred at 25°C for 12 h. The reaction solution was concentrated and poured into a large amount of ice ether for sedimentation. The mixture was centrifuged and vacuum-dried to obtain PAE (SS)-PEG5k-NHS. PAE (SS)-PEG5k-NHS and Tf were dissolved in DMSO, stirred at 25°C for 12 h. Then, the mixture was filtered, dialyzed, and lyophilized to obtain Tf-PEG-PAE (SS).

### Preparation of the Drug-loaded Nanocarrier

TMZ and Tf-PEG-PAE(SS) were dissolved in organic solvent and vortexed strenuously at 25°C for 1 h, and then, the mixture solution was stirred for 30 min at 25°C and dialyzed for 24 h. A certain amount of siEGFR was blended with the Tf-PEG-PA (SS)/TMZ micelle by stirring for 60 s and then allowed for reaction for 1 h at 25°C. Using this method, diverse siEGFR-loaded nanocomplexes of Tf-PEG-PAE(SS)/TMZ@siEGFR were produced through different ratios of N/P.

### Characterization of the Drug-Loaded Nanocarrier

^1^H NMR spectra were recorded using a Bruker 400 MHz spectrometer. Particle sizes and zeta potentials were measured using a Malvern Zetasizer Nano ZS90 apparatus at 25°C (Malvern Instruments, Malvern, UK). The TMZ release profile was analyzed by high-performance liquid chromatography (HPLC) (Vanquish Duo HPLC, MA, America).

### Gel Retardation Assay

The binding ability of siEGFR with Tf-PEG-PAE(SS)/TMZ micelle was studied through agarose gel electrophoresis. In detail, gel electrophoresis was executed using 2% (w/v) agarose gel in TAE buffer with 0.5 μg/ml of EtBr. Tf-PEG-PAE(SS)/TMZ@siEGFR at diverse N/P ratios (0.5, 1.0, 2.0, 5.0, 10.0, 15.0, and 20.0) and 6× DNA loading buffer were prepared and mixed in the ratio of 5:1 for electrophoresis. The total amount of samples added per well was 20 μl, and then, the process was carried out at 120 V for 10 min. The result was detected using the DNR Bio-Imaging System.

### Cellular Uptake Assay

Cy5-TMZ and FAM-siEGFR were managed as a fluorescent probe to reflect the intracellular uptake of nanocarriers. In the experiment, 1 × 10^5^ U87 cells were seeded in 6-well plates in each well and incubated for 24 h. Then, the cells were incubated for 4 h in the FBS-free medium treating with the nanocarriers at a TMZ concentration of 30 nM. For the free TfR inhibition assay, the cells were pretreated with Tf (2 mg/l) for 1 h before the targeting nanocarrier was added to the culture medium. Then, the targeting efficiency of the Tf-PEG-PAE(SS)/TMZ@siEGFR was assessed using a fluorescence microscope and flow cytometry. The intracellular distribution of the Tf-PEG-PAE(SS)/TMZ@ siEGFR was further evaluated by confocal laser scanning microscopy (CLSM).

### *In Vitro* Cytotoxicity

The MTT assay on U87 cells was conducted to evaluate the nanoparticle cytotoxicity (Hua et al., 2018). U87 cells were seeded in 96-well plates at a density of 1 × 10^4^ cells/well and incubated for 24 h. Then, they were incubated with the following reagents for 72 h: 1) PEG-PAE (SS), 2) Tf-PEG-PAE (SS), 3) Tf-targeted nanocarrier loading TMZ and siRNA (Tf-PEG-PAE(SS)/TMZ@siEGFR and Tf-PEG-PAE(SS)/TMZ@siRNA^nc^), and 4) non-Tf-targeted nanocarrier loading TMZ and siRNA (PEG-PAE(SS)/TMZ@ siEGFR and PEG-PAE(SS)/TMZ@siRNA^nc^). The siRNA concentration was 20 nM in each well. Then, 100 μl of 0.5 mg/ml MTT was added to each well for incubation at 37°C. After 4 h, the MTT medium was removed, and DMSO was added. Then, the optical density was detected by using spectrophotometric analysis at 540 nm.

### Clonogenic Assay

To evaluate the effects of Tf-PAE-PEG/TMZ@siEGFR on the chemosensitivity of U87 cells, the colony formation assay was used. U87 cells were seeded in 6-well plates at a density of 3×10^3^/well and incubated for 24 h. The cells were treated with different groups (PBS, TMZ@siEGFR, PEG-PAE(SS)/TMZ@siEGFR, and Tf-PEG-PAE(SS)/TMZ@siEGFR) at a TMZ concentration of 30 nM for 4 h. After 4 h, the existed DMEM medium was displaced by a fresh DMEM medium and then cultured for 14 days until the colonies exceeded.

### Cell Apoptosis

U87 cells were seeded in 6-well plates at a density of 1 × 10^4^ cells/well and incubated for 24 h. Then, the cells were cultured for 48 h in the FBS-free medium treated with PBS, TMZ@siEGFR, PEG-PAE(SS)/TMZ@siEGFR, or Tf-PEG-PAE(SS)/TMZ@siEGFR. Subsequently, the cells were trypsinized, collected, washed, resuspended, and stained with Annexin V-FITC/PI for 30 min at 37°C. Last, the stained cells were analyzed using a flow cytometer (BD FACSCalibur, NJ, America) (Zhan et al., 2019).

### EdU Assay

U87 cells were seeded in 96-well plates at a density of 1 × 10^5^ cells/well and incubated for 24 h. After treating with five groups of drugs: PBS, free TMZ, TMZ@siEGFR, PEG-PAE(SS)/TMZ@siEGFR, or Tf-PEG-PAE(SS)/TMZ@siEGFR, they were incubated in the medium for 4 h. Then, 50 μM of EdU was added to the treated cells for 2 h at room temperature and washed with PBS three times to elute the EdU reagent. Subsequently, the cells were fixed with 4% paraformaldehyde for 30 min and then incubated with 0.5% Triton X-100 for 10 min at room temperature. Under the condition of avoiding light at room temperature, 1 × Apollo^®^ dye solution was used to react with cells for 30 min, and 1 x Hoechst 33,342 was dyed for another 30 min. Finally, the stained cells were washed with PBS three times, and the staining results were observed using a fluorescence microscope.

### Quantitative Real-Time Polymerase Chain Reaction (qRT-PCR)

qRT-PCR was used to assess the expression levels of EGFR mRNA in U87 cells. First, the total RNA was extracted using the TRIzol reagent, and DNase I was used to remove the possible DNA contamination by digesting the extracted RNA. Second, the RNA was purified again using the TRIzol reagent, and the cDNA was synthesized using the reverse transcription kit. Finally, the quantitative analysis of cDNA was calculated using a qRT-PCR machine (Eppendorf AG, Hamburg, Germany).

### Western Blotting Analysis

The ability of the siRNA delivered by the Tf-PEG-PAE(SS) polymer in silencing the EGFR gene expression was evaluated by Western blotting. U87 cells (5 × 10^5^) were seeded in a 6-well plate and cultured at 37°C in 5% CO_2_ for 24 h. Subsequently, the cells were cultured with PBS, free TMZ, TMZ@siEGFR, PEG-PAE(SS)/TMZ@siEGFR, or Tf-PEG-PAE(SS)/TMZ@siEGFR at a dose of 1 μg/ml siEGFR for 24 h. The transfected cells were washed with PBS, and then harvested using the lysis buffer. Then, the cell lysates were cultured for 30 min at 4°C and centrifuged at a speed of 12,000 rpm for 15 min. The protein concentrations were calculated through the BCA protein assay. The proteins were separated with SDS polyacrylamide gel and transfected onto polyvinylidene difluoride membranes. Next, the membranes were blocked with skimmed milk in TBST buffer and incubated with the anti-EGFR antibody at 4°C overnight. The following day, the membranes were incubated with secondary antibodies at 37°C for 1 h. Last, the membranes were washed and exposed in a dark room.

### U87 Glioma Model

Glioma cells (U87 cells) were transformed with the luciferase gene (U87-luci). The brain glioma model was established by the stereotactic implantation of U87-luci cells (Shi et al., 2020). After that, 1 × 10^5^ U87-luci cells in 5 μl of L15 medium were injected for 10 min and allowed to stay for 5 min into the right striatum with microsyringe before removing. Then, the burr hole was sealed off with bone wax. After 10 days, the intensity of luci-fluorescence was observed using an *in vivo* imaging system to confirm the successful construction of the glioma model. Subsequently, the nude mouse glioma models were randomly divided into five groups (n = 10 in each group): PBS, free TMZ, TMZ@siEGFR, PEG-PAE(SS)/TMZ@siEGFR, and Tf-PEG-PAE(SS)/TMZ@siEGFR.

### *In Vivo* Distribution

On day 20, the nude mouse glioma models were checked for their coincident size. Subsequently, these mice were stochastically separated into six groups (n = 3 in each group): Cy5-labeled TMZ (free TMZ^Cy5^), FAM-labeled siEGFR (free siEGFR^FAM^), PEG-PAE(SS)/TMZ^Cy5^@siEGFR, PEG-PAE(SS)/TMZ@siEGFR^FAM^, Tf-PEG-PAE(SS)/TMZ^Cy5^@siEGFR, and Tf-PEG-PAE(SS)/TMZ@siEGFR^FAM^. Each group was injected with doses of TMZ^Cy5^, siEGFR^FAM^, TMZ^Cy5^, or siEGFR^FAM^ co-loaded NPs (TMZ^Cy5^ = 2 mg/kg and siEGFR^FAM^ = 1 mg/kg) *via* the tail vein, respectively. After 4 h, the mice were killed, and the major organs (the brain, heart, liver, spleen, lung, and kidney) were isolated and imaged through the Living Image System.

### *In Vivo* Anticancer Efficacy

*In vivo* fluorescence imaging, survival time, and weight sizes were utilized to reflect the therapeutic outcomes of different groups (Hua et al., 2018). The nude mouse glioma models were stochastically separated into five groups (n = 10) and injected with PBS, free TMZ, TMZ@siEGFR, PEG-PAE(SS)/TMZ@siEGFR, or Tf-PEG-PAE(SS)/TMZ@siEGFR on days 12, 14, and 16 after implantation, respectively. TMZ was treated with a dose of 3 mg/kg, and siEGFR was treated with a dose of 1 mg/kg. The images of the glioma-bearing mice were taken on days 20 and 30 using the *in vivo* imaging system, and the Live Image Software was used to analyze the bioluminescence signals (PerkinElmer, MA, United States). In the entire process of the experiment, we recorded the survival time and the weight changes of the mice in each group every 2 days.

### Toxicity Study

After the last 24 h treatment, the mice were killed to assess the organ safety. The major organs including the heart, liver, spleen, lung, and kidney were collected and made into sections stained with hematoxylin and eosin (H&E). For further evaluation of the pathological changes, the serum was collected to reflect the levels of ALT, AST, BUN, and CREA.

### Statistical Analysis

All experimental data were shown as mean ± SD and executed at least three times. The data were analyzed using one-way analysis of variance and two-tailed Student’s t-tests. *p* < 0.05 (*), *p* < 0.01 (**), or *p* < 0.001 (***) was considered to be statistically significant at different levels.

## Results and Discussion

### Synthesis and Characterization of Tf-PEG-PAE(SS)/TMZ@siEGFR

**FIGURE 2:**
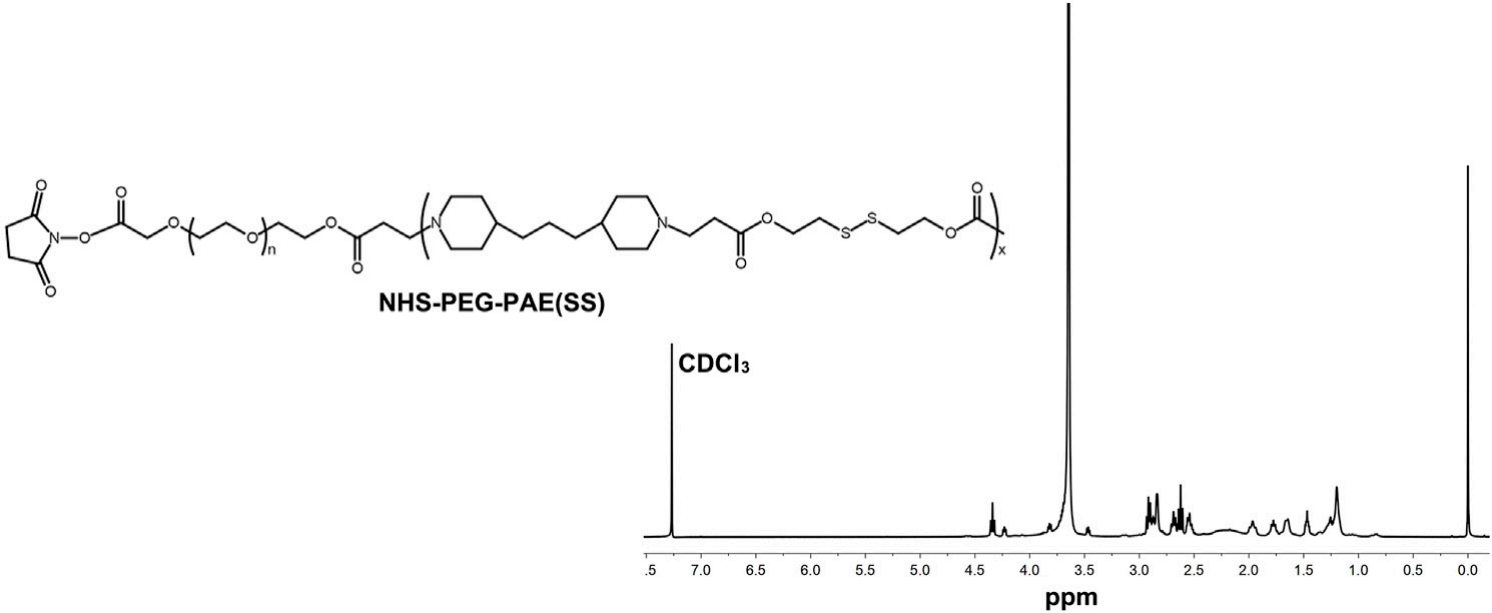
^1^H NMR spectra of NHS-PEG-PAE(SS).

A pH-sensitive copolymer NHS-PEG-PAE(SS) was synthesized *via* a multistep reaction. The composition of the NHS-PEG-PAE (SS) was further confirmed by ^1^H NMR analysis. The ^1^H NMR spectrum explicitly exhibited the characteristic resonance peaks of PAE and PEG blocks, respectively (Figure 2). Next, the Tf-PEG-PAE(SS)/TMZ@siEGFR was constructed successfully (Figure 1).

**FIGURE 3:**
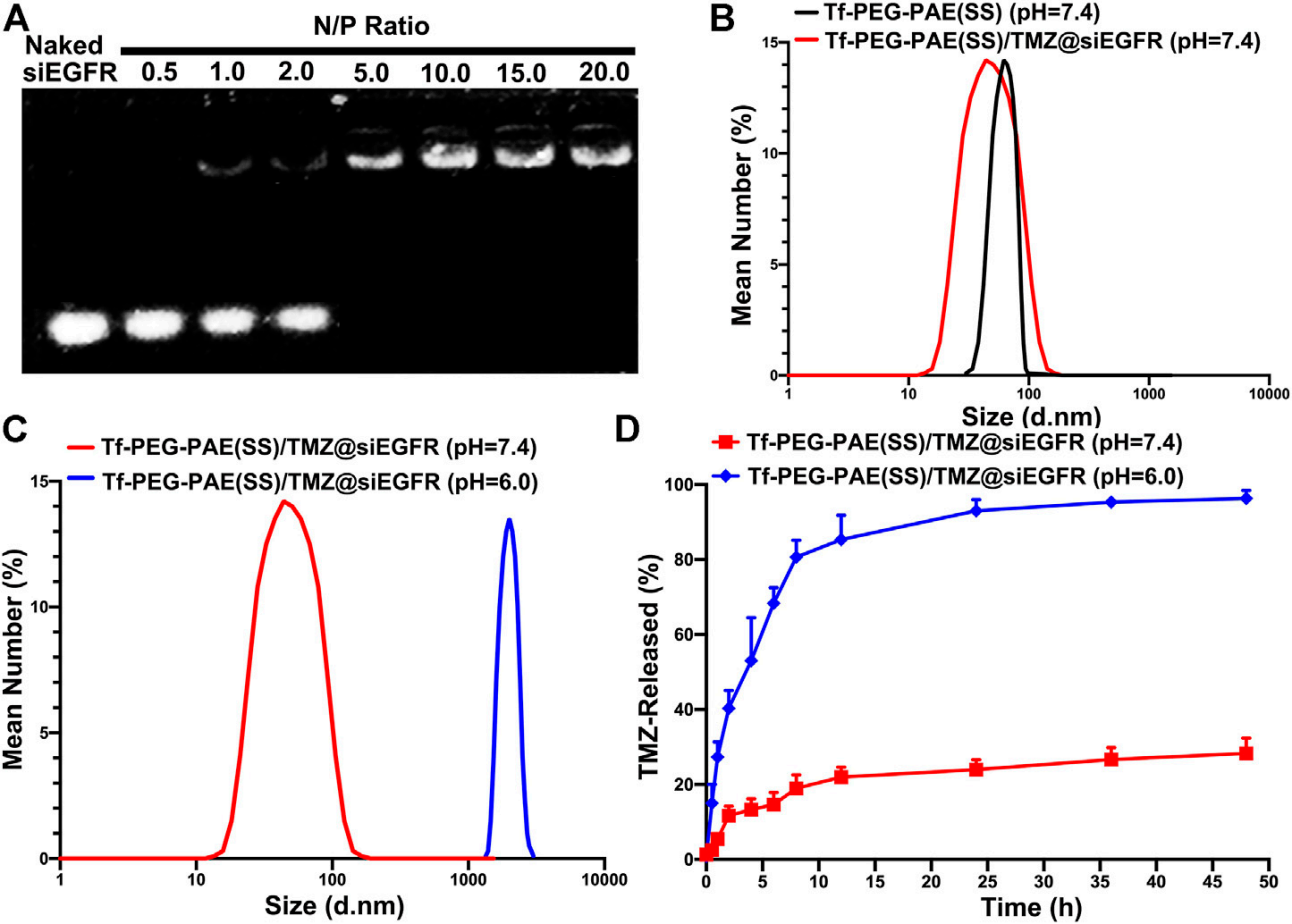
Characterization of the pH-sensitive Tf-PEG-PAE(SS)/TMZ@siEGFR. **(A)** siEGFR binding ability at numerous N/P ratios. **(B–C)** Average size of Tf-PEG-PAE (SS) and Tf-PEG-PAE(SS)/TMZ@siEGFR at pH 7.4 and pH 6.0. **(D)** *In vitro* drug release of Tf-PEG-PAE(SS)/TMZ@siEGFR at pH 7.4 and pH 6.0 (Mean ± SD, n = 3).

As shown in Figure 3A, Tf-PEG-PAE(SS)/TMZ showed an intense ability in binding siEGFR at a N/P ratio of 5: 1 in the gel. According to the TEM image, Tf-PEG-PAE(SS)/TMZ@siEGFR owned well-defined spherical morphologies with an average particle size around 80 nm at pH 7.4, which was consistent with the DLS determination. According to dynamic light scattering (DLS) detection, Tf-PEG-PAE(SS) and Tf-PEG-PAE(SS)/TMZ@siEGFR possessed a diameter of 95.34 ± 3.25 nm and 80.29 ± 3.14 nm, respectively (Figure 3B). In a sharp contrast, when the Tf-PEG-PAE(SS)/TMZ@siEGFR solution was adjusted to a pH value of 6.0, the average particle size increased significantly, which was due to the destabilization and conformational change of the Tf-PEG-PAE(SS) under acid conditions (Figure 3C). Tf-PEG-PAE(SS)/TMZ@siEGFR was predicted to have a great potential for pH-responsive controlled drug release. Therefore, the TMZ released from Tf-PEG-PAE(SS)/TMZ@siEGFR was investigated at pH 7.4 and 6.0 *in vitro*. As shown in Figure 3D, the TMZ release was clearly quickened at pH 6.0 (blue squares), suggesting that Tf-PEG-PAE(SS)/TMZ@siEGFR selectively dissociates and releases under acid conditions. However, no significant drug release was found at pH 7.4 (red squares).

### Cellular Uptake Studies

**FIGURE 4:**
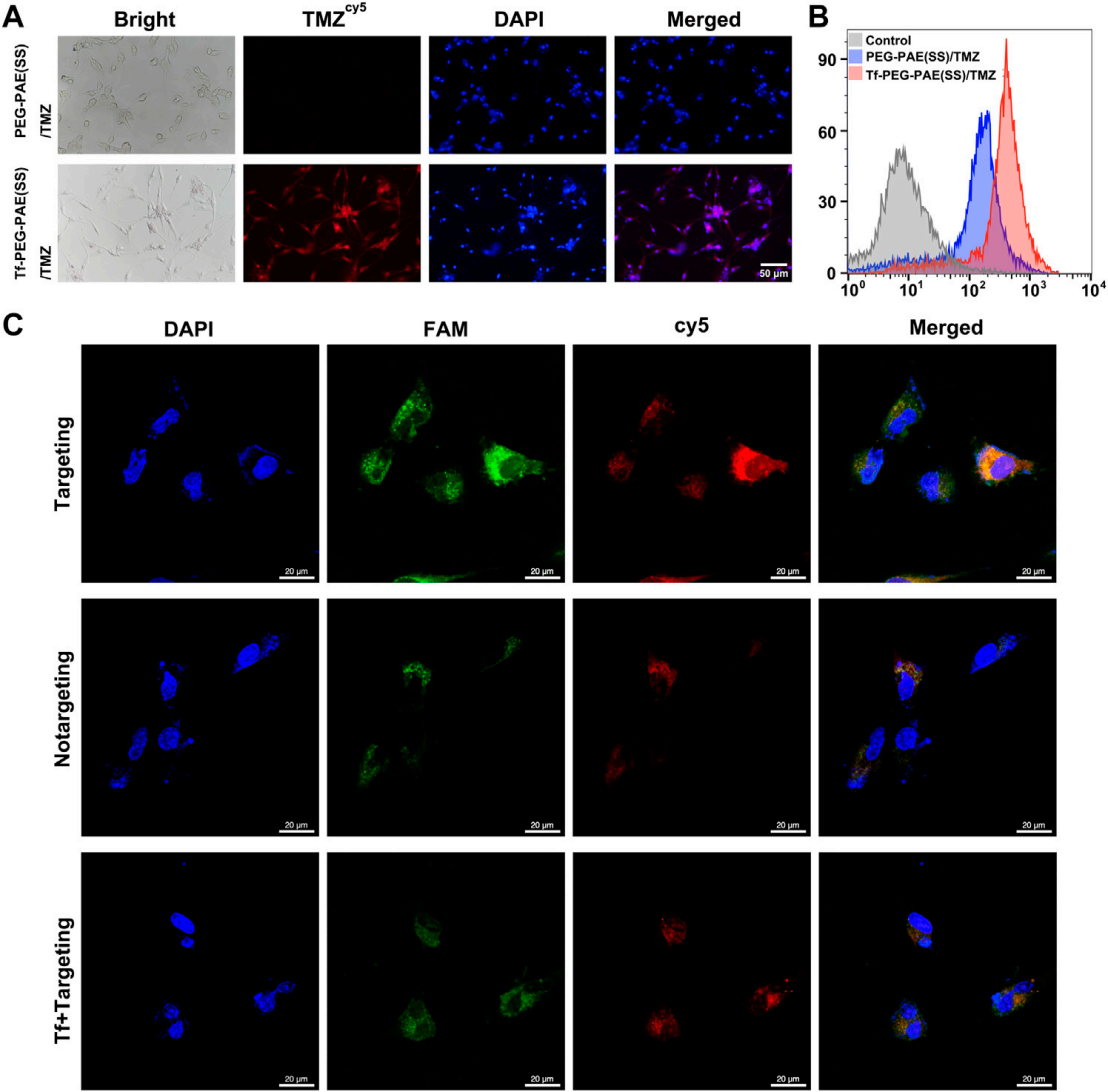
**(A)** Intracellular release of TMZ^cy5^ from PEG-PAE(SS)/TMZ^cy5^ and Tf-PEG-PAE(SS)/TMZ^cy5^, Scale bar: 50 μm. **(B)** Drug fluorescence of PEG-PAE (SS)/TMZ^cy5^ and Tf-PEG-PAE (SS)/TMZ^cy5^ in cells was analyzed by flow cytometry. **(C)** CLSM images of U87 cells incubated with Tf-PEG-PAE(SS)/TMZ^cy5^@siEGFR^FAM^ (targeting), PEG-PAE(SS)/TMZ^cy5^@siEGFR^FAM^ (nontargeting), and Tf-PEG-PAE(SS)/TMZ^cy5^@siEGFR^FAM^ with 2 mg/l Tf (Tf + targeting). Blue, green, red, and orange fluorescence indicate DAPI (nuclei), FAM (siEGFR), Cy5 (TMZ), and the overlapping of FAM and Cy5, respectively. Scale bar: 20 μm.

In glioma therapy, TMZ and siEGFR encapsulated within NPs must be released into the glioma cells in order to exhibit a synergistic anticancer effect. Researchers have modified a targeting ligand-transferrin (Tf) liposome to specifically recognize transferrin receptors (TfR), increasing brain drug delivery (Li et al., 2021). In this study, Tf was modified on the surface of PEG-PAE(SS)/TMZ@siEGFR to increase the BBB penetrating efficiency and the glioma distribution of drugs. To test the intracellular efficiency of TMZ and siEGFR in Tf-PEG-PAE(SS)/TMZ@siEGFR, Cy5-labeled TMZ (TMZ^cy5^) that radiates red fluorescence and FAM-labeled siEGFR (siEGFR^FAM^) that radiates green fluorescence were loaded into the nanocarrier, and the human glioma cell line U87 was used for testing after 4 h incubation. The fluorescence microscopy results demonstrated that cells incubated with Tf-PEG-PAE(SS)/TMZ^cy5^ displayed much stronger TMZ^cy5^ fluorescence than the nontargeting PEG-PAE(SS)/TMZ^cy5^ group (Figure 4A). Similarly, the flow cytometry analysis indicated that the TMZ^cy5^ in cells rose obviously when they were incubated with Tf-PEG-PAE(SS)/TMZ^cy5^ rather than PEG-PAE(SS)/TMZ^cy5^ (Figure 4B). These results suggested that the Tf modification enhanced the endocytosis of Tf-PEG-PAE(SS)/TMZ@siEGFR. Moreover, to further estimate the targeting effect of Tf-PEG-PAE(SS)/TMZ@siEGFR, the CLSM was applied to show the drug fluorescence distribution in glioma cells directly. The distributions of TMZ^cy5^ fluorescence and siEGFR^FAM^ fluorescence were almost within the cytoplasm and around the nuclei. As a result of red and green fluorescence overlapping in the merged image, orange stains were generated (Figure 4C). In front of 2 mg/l transferrin, the endocytosis level of Tf-PEG-PAE(SS)/TMZ^cy5^@siEGFR^FAM^ was significantly weakened. Even the ratio of TMZ^cy5^ and siEGFR^FAM^ positive cells was nearly wakened to the same level as that in cells treated with PEG-PAE(SS)/TMZ^cy5^@siEGFR^FAM^. Thus, Tf mediated an effective cellular uptake of nanoparticles, accelerating the co-delivery of TMZ and siEGFR into the U87 cells.

### *In Vitro* Synergistic Anticancer Effect

**FIGURE 5:**
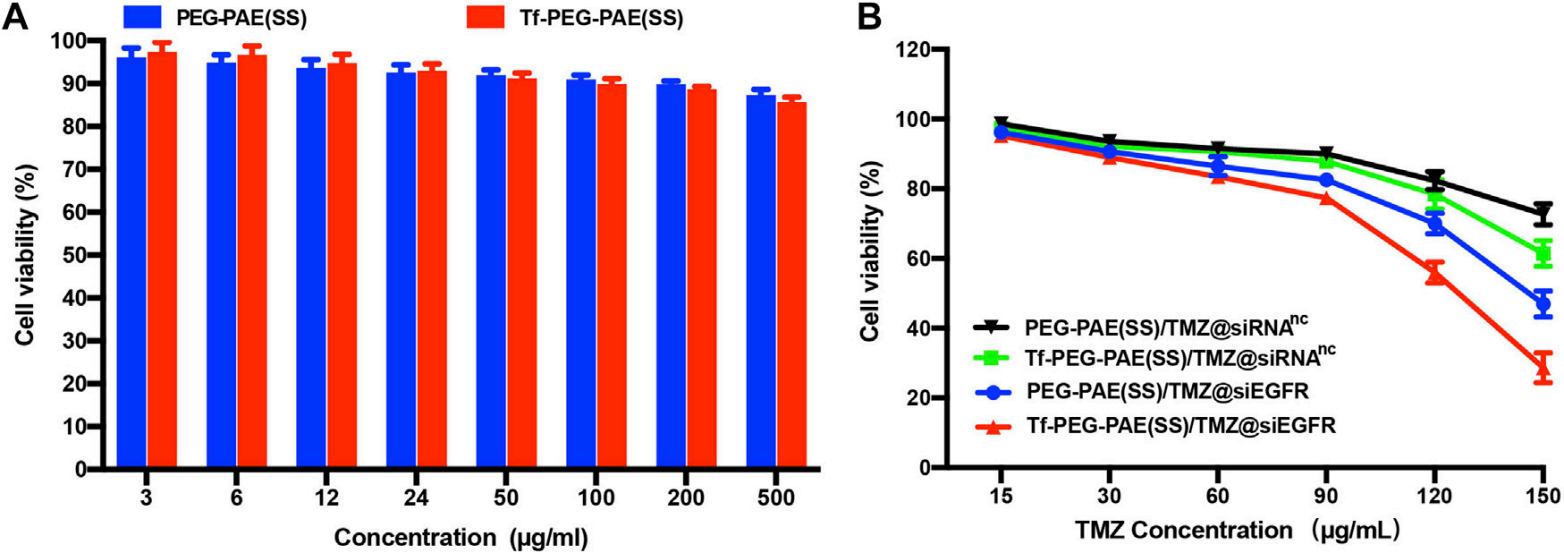
**(A)** *In vitro* cytotoxicities of Tf-PEG-PAE(SS) and PEG-PAE(SS) at diverse concentrations in U87 cells. **(B)** *In vitro* cytotoxicities of PEG-PAE(SS)/TMZ@siRNA^nc^, Tf-PEG-PAE(SS)/TMZ@siRNA^nc^, PEG-PAE(SS)/TMZ@siEGFR, and Tf-PEG-PAE(SS)/TMZ@siEGFR at diverse concentrations in U87 cells.

The cytotoxicities of Tf-PEG-PAE(SS) and PEG-PAE(SS) were investigated using the MTT assay. U87 cells incubated with Tf-PEG-PAE(SS) and PEG-PAE(SS) showed above 85% viability even at a high concentration of 500 μg/ml, revealing a low cytotoxicity, which was significant for the follow-up experiments *in vivo* (Figure 5A). For U87 cells dealing with the TMZ-carried nanocomposite, a negative correlation was monitored between the TMZ concentration and cell survival. In addition, Tf-PEG-PAE(SS)/TMZ@siEGFR displayed substantially higher cytotoxicity than PEG-PAE(SS)/TMZ@siRNA^nc^, Tf-PEG-PAE(SS)/TMZ@siRNA^nc^, and PEG-PAE(SS)/TMZ@siEGFR (Figure 5B). For instance, when the TMZ concentration reached 150 μg/ml, U87 cells incubated with Tf-PEG-PAE(SS)/TMZ@siEGFR displayed 28.67% viability, while cells incubated with other micelles exhibited >46.93% viability. The acid condition could induce the degradation of the hydrophobic PAE core to release TMZ and siEGFR by increasing the chemosensitivity of temozolomide-resistant glioma cells, enhancing the cell apoptosis.

**FIGURE 6:**
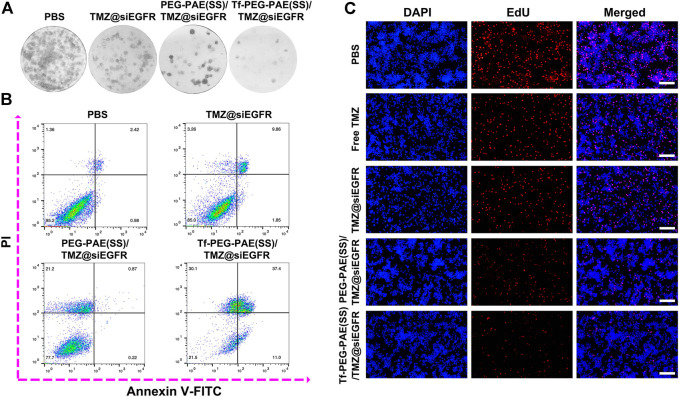
**(A)** Clonogenic formation of U87 cells treated with PBS, TMZ@siEGFR, PEG-PAE(SS)/TMZ@siEGFR, or Tf-PEG-PAE(SS)/TMZ@siEGFR. **(B)** Analysis of apoptotic U87 cells cultured with PBS, TMZ@siEGFR, PEG-PAE(SS)/TMZ@siEGFR, or Tf-PEG-PAE(SS)/TMZ@siEGFR by flow cytometry. **(C)** Representative images of EdU assays cultured with PBS, free TMZ, TMZ@siEGFR, PEG-PAE(SS)/TMZ@siEGFR, or Tf-PEG-PAE(SS)/TMZ@siEGFR.

The colony-forming assay and flow cytometry were conducted to calculate whether there was a synergistic effect of TMZ and siEGFR on the instigating apoptosis of U87 cells. As indicated by the colony formation capacity, Tf-PEG-PAE(SS)/TMZ@siEGFR had a chemotherapy sensitization function in U87 cells (Figure 6A). As observed from the Annexin V-FITC and PI dual staining detection assay, the control cells without treatment showed an extremely low apoptosis rate of 3.4%. By contrast, the apoptosis level of cells treated with Tf-PEG-PAE(SS)/TMZ@siEGFR was up to 48.4%, which was higher than that of TMZ@siEGFR (11.7%) and PEG-PAE(SS)/TMZ@siEGFR (1.1%) treatment groups (Figure 6B). To further assess the anticancer performance, the EdU experiment was employed. Tf-PEG-PAE(SS)/TMZ@siEGFR induced the lowest cell proliferation among all groups (Figure 6C). These results revealed that the co-delivery of TMZ and siEGFR showed a synergistic anticancer effect in accelerating cellular apoptosis.

### Gene Silencing Capability

**FIGURE 7:**
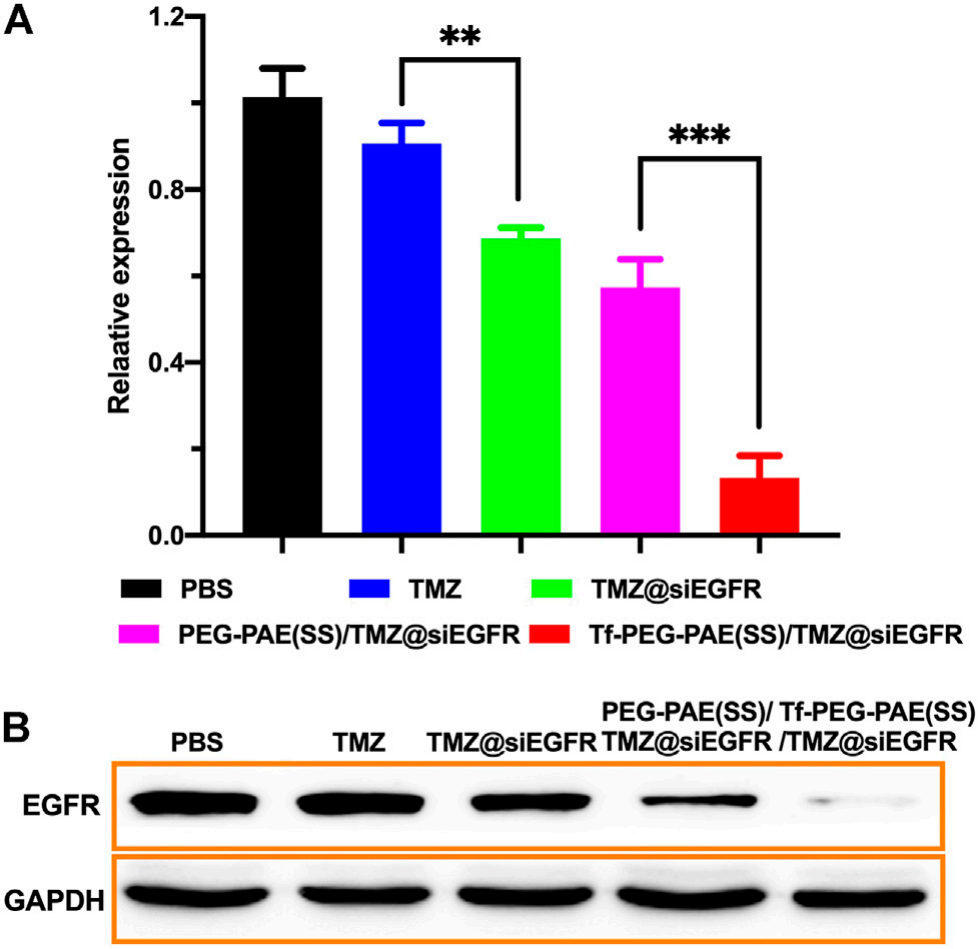
*In vitro* gene silencing capability. **(A)** qRT-PCR analysis results of EGFR mRNA expression with different treatments. **(B)** Western blot analysis of the EGFR protein expression with different treatments. (Mean ± SD, n = 3), ^∗∗^ *p* < 0.01, and ^∗∗∗^ *p* < 0.001.

The overexpression of EGFR was found in GBM, which may result in strengthening proliferation and restraining apoptosis of glioma cells (Fang et al., 2021). Hence, knocking down of the EGFR expression would offer a way to improve the sensitivity of TMZ (Tsai et al., 2019). In this study, we used qRT-PCR and Western blotting to assess the transcription and translation of EGFR, respectively. As revealed by qRT-PCR, the treatment with Tf-PEG-PAE(SS)/TMZ@siEGFR obviously reduced the expression of EGFR at the mRNA level (86.7%), as compared to free TMZ (9.3%), TMZ@siEGFR (31.3%), and PEG-PAE(SS)/TMZ@siEGFR (42.7%) (Figure 7A). In the Western blotting experiment, the result was similar to the qRT-PCR data (Figure 7B). The qRT-PCR and Western blotting results both demonstrated that TMZ and siEGFR co-delivered by the Tf-PEG-PAE(SS)/TMZ@siEGFR showed great ability in silencing EGFR.

### Biodistribution of Tf-PEG-PAE(SS)/TMZ@siEGFR *In Vivo*

**FIGURE 8:**
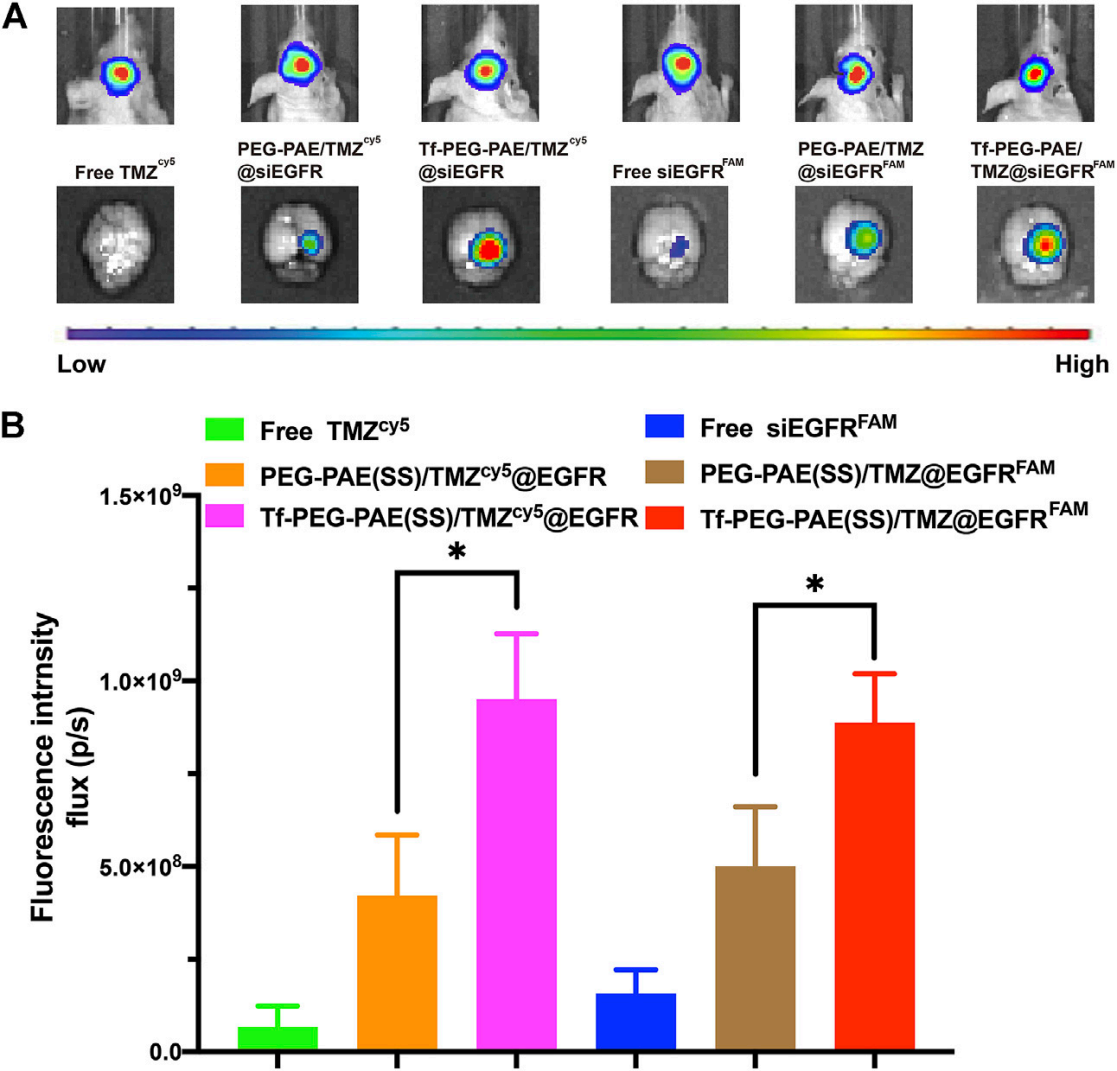
Distribution of TMZ and siEGFR in glioma. **(A)** Biofluorescence imaging of gliomas with the similar size, 4 h after intravenous injection of mice with either free TMZ^cy5^, free siEGFR^FAM^, PEG-PAE(SS)/TMZ^cy5^@siEGFR, PEG-PAE(SS)/TMZ@siEGFR^FAM^, Tf-PEG-PAE(SS)/TMZ^cy5^@siEGFR, and Tf-PEG-PAE(SS)/TMZ@siEGFR^FAM^. **(B)** Quantitative analysis of TMZ and siEGFR fluorescence intensity in glioma. (Mean ± SD, n = 3) and ^∗^ *p* < 0.05.

The targeting ability of Tf-PEG-PAE(SS)/TMZ@siEGFR in striding over the BBB and permeating into GBM is vital for the synergistic therapy. In this study, we applied an *in vivo* fluorescence imaging technique to examine the distribution of Tf-PEG-PAE(SS)/TMZ^cy5^@siEGFR or Tf-PEG-PAE(SS)/TMZ@siEGFR^FAM^ after intravenous injection into models. An orthotopic implantation model was established by U87-Luci glioma cells in nude mice. On day 10 , the luciferase signal was detected, confirming the presence of a brain glioma with approximately the same volume (Figure 8A). Free TMZ^cy5^, free siEGFR^FAM^, PEG-PAE(SS)/TMZ^cy5^@siEGFR, PEG-PAE(SS)/TMZ@siEGFR^FAM^, Tf-PEG-PAE(SS)/TMZ^cy5^@siEGFR, and Tf-PEG-PAE(SS)/TMZ@siEGFR^FAM^ were then injected *via* the tail vein, respectively. After 4 h, gliomas were excised and observed using an *in vivo* fluorescence imaging system (Figures 8A,B). Compared with free TMZ^cy5^ and free siEGFR^FAM^, PEG-PAE(SS)/TMZ^cy5^@siEGFR or PEG-PAE(SS)/TMZ@siEGFR^FAM^ exhibited the stronger cy5 or FAM fluorescence in glioma, revealing that enhanced permeability and retention effect (EPR) make nanocarriers tend to accumulate in the tumor tissue passively. Compared with PEG-PAE(SS)/TMZ^cy5^@siEGFR and PEG-PAE(SS)/TMZ@siEGFR^FAM^, Tf-PEG-PAE(SS)/TMZ^cy5^@siEGFR or Tf-PEG-PAE(SS)/TMZ@siEGFR^FAM^ showed the strongest fluorescence, indicating Tf-modified nanocarrier promoted TMZ and siEGFR to cross BBB and target glioma forwardly.

**FIGURE 9:**
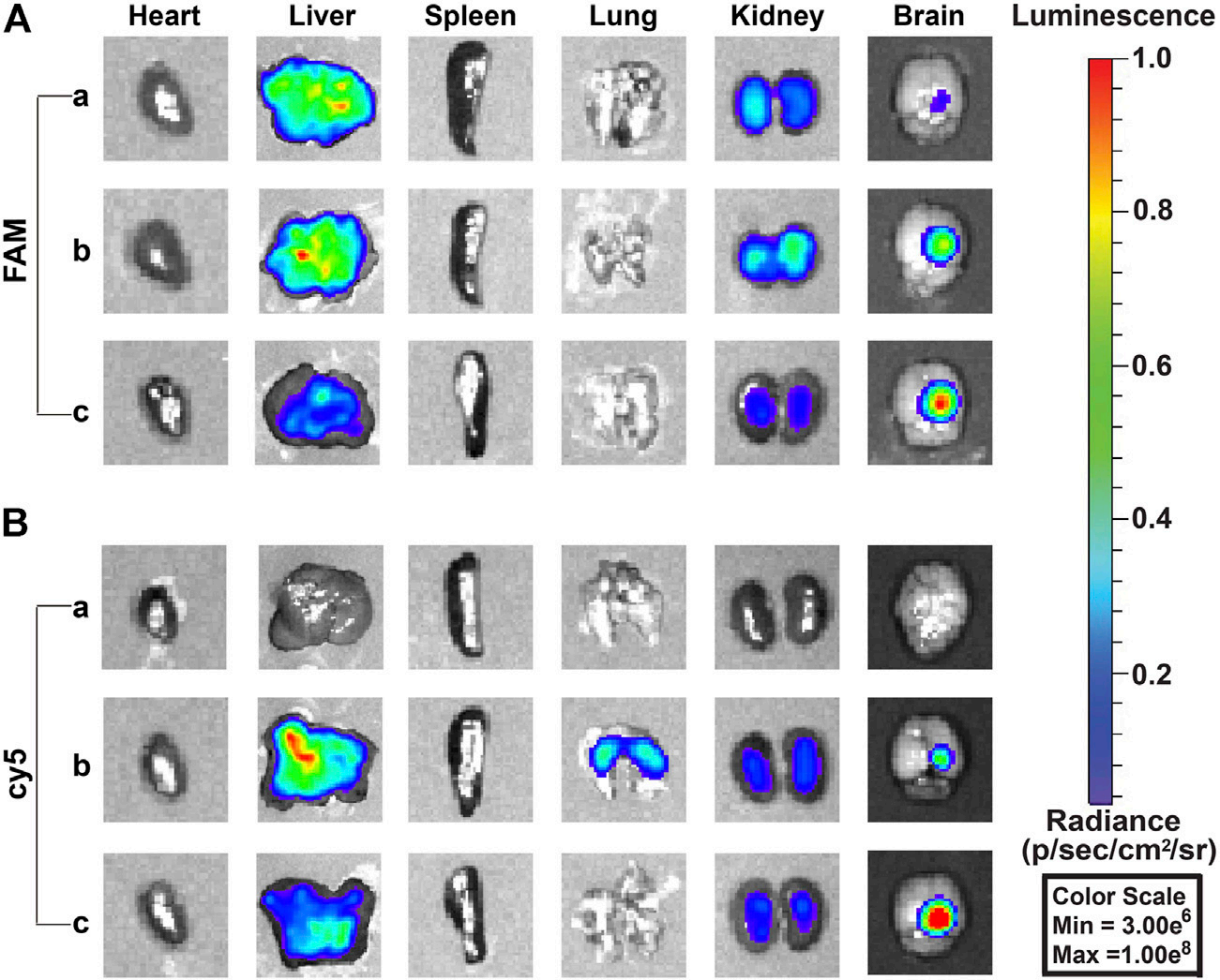
Fluorescence images of siEGFR^FAM^ and TMZ^cy5^ in organs after intravenous injection of complex nanoparticles (n = 3). **(A)** Fluorescence image of the FAM channel: (a) free siEGFR^FAM^, (b) PEG-PAE(SS)/TMZ@siEGFR^FAM^, and (c) Tf-PEG-PAE(SS)/TMZ@siEGFR^FAM^. **(B)** Fluorescence image of cy5 channel: (a) free TMZ^cy5^, (b) PEG-PAE(SS)/TMZ^cy5^@siEGFR, and (c) Tf-PEG-PAE(SS)/TMZ^cy5^@siEGFR.

The biodistribution *in vivo* was examined after the injection of free TMZ^cy5^, free siEGFR^FAM^, PEG-PAE(SS)/TMZ^cy5^@siEGFR, PEG-PAE(SS)/TMZ@siEGFR^FAM^, Tf-PEG-PAE(SS)/TMZ^cy5^@siEGFR, and Tf-PEG-PAE(SS)/TMZ@siEGFR^FAM^ by the tail vein; the biodistributions of TMZ^cy5^ and siEGFR^FAM^ in glioma-bearing mice are shown in Figure 9. The TMZ concentration level in glioma is dramatically higher in the Tf-PEG-PAE(SS)/TMZ^cy5^@siEGFR group than that in the free TMZ^cy5^ or PEG-PAE(SS)/TMZ^cy5^@siEGFR group (Figure 9B). In addition, the fluorescence strength in all anatomized organs in the Tf-PEG-PAE(SS)/TMZ^cy5^@siEGFR group was lower than that in the other groups. In the FAM channel, few fluorescent signals were checked in the free siEGFR^FAM^ group, revealing that the free siEGFR barely gets effective EGFR interference without an expedient delivery platform. The FAM intensity in the glioma-bearing mice injected with Tf-PEG-PAE(SS)/TMZ@siEGFR^FAM^ is much stronger than that in the PEG-PAE(SS)/TMZ@siEGFR^FAM^ group (Figure 9A). These experimental results prompted that the Tf decoration exerted a crucial function in this nano transportation system, in line with the previous finding that the Tf could enhance the drug accumulation in tumors.

### *In Vivo* Anticancer Efficacy

**FIGURE 10:**
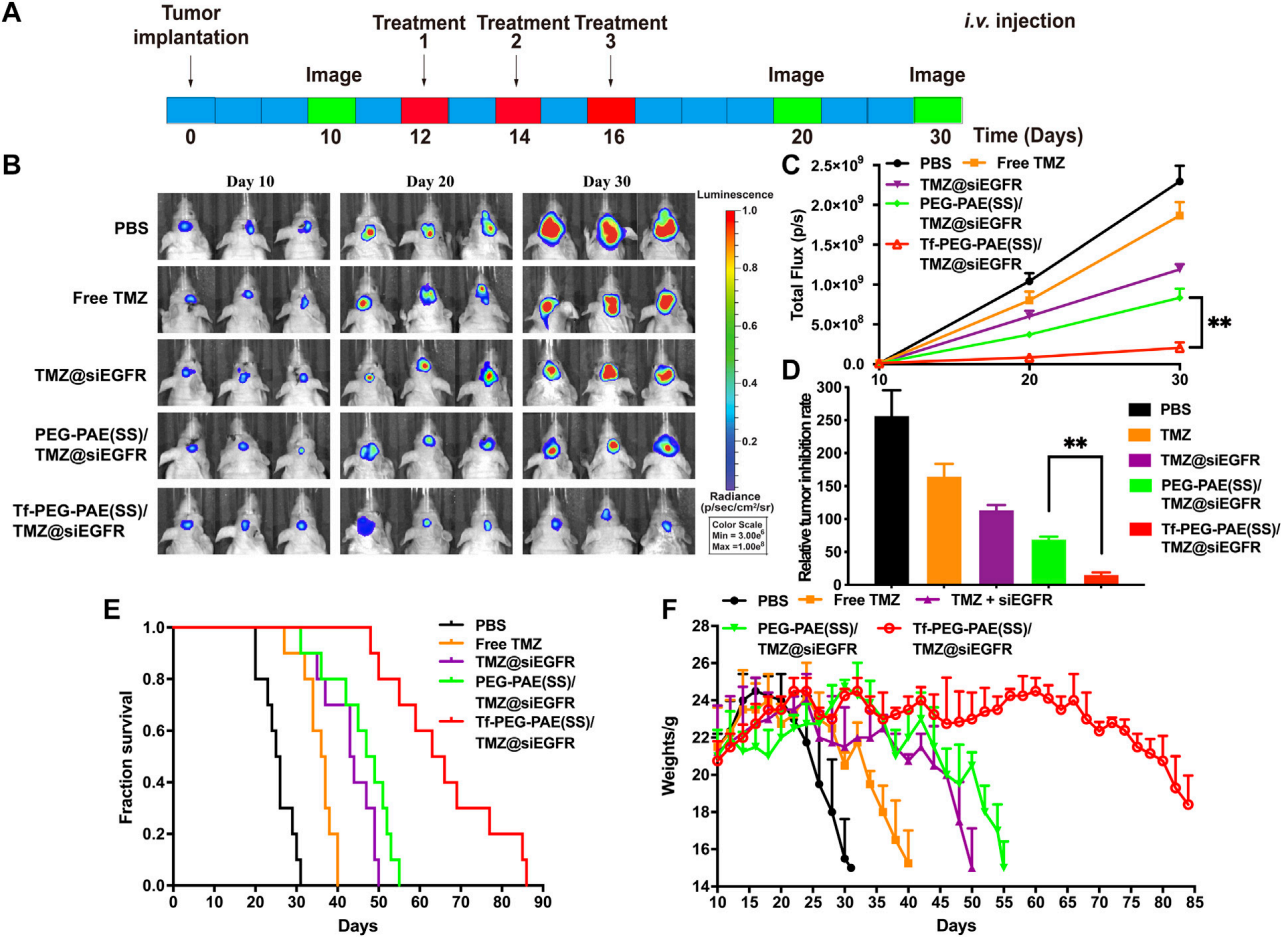
Synergistic anticancer activity of complex nanoparticles on the U87-Luci glioma mouse model. **(A)** U87-Luci-bearing mice received five injections of PBS, free TMZ, TMZ@siEGFR, PEG-PAE(SS)/TMZ@siEGFR, and Tf-PEG-PAE(SS)/TMZ@siEGFR. **(B)** Bioluminescence signal change of different groups. **(C)** Quantification of the tumor bioluminescence signal (n = 10), ^∗∗^ *p* < 0.01. **(D)** Relative tumor inhibitory rate for each group, ^∗∗^ *p* < 0.01. **(E)** Kaplan–Meier survival curves for the mice (n = 10). **(F)** Body weights changed (n = 10).

The orthotopic implantation models were applied to assess the therapeutic effect of Tf-PEG-PAE(SS) carrying both TMZ and siEGFR *in vivo*. The mice were randomly divided into five groups (n = 10), and treated with PBS, free TMZ, TMZ@siEGFR, PEG-PAE(SS)/TMZ@siEGFR, or Tf-PEG-PAE(SS)/TMZ@siEGFR *via* tail intravenous injection three times during the experimental process (the dose of TMZ = 3 mg/kg and siEGFR = 1 mg/kg) (Figure 10A). On days 10, 20, and 30, the tumor bioluminescence intensity was monitored to evaluate its growth. In the PBS group as a negative control, tumor grew rapidly (glioma inhibition rate of 256.5). By contrast, in treating groups, the glioma inhibition rates of free TMZ, TMZ@siEGFR, PEG-PAE(SS)/TMZ@siEGFR, and Tf-PEG-PAE(SS)/TMZ@siEGFR on day 30 were 164.3, 113.3, 69.2, and 15.3, respectively (Figures 10B,D). According to these results, Tf-PEG-PAE(SS)/TMZ@siEGFR showed the highest antitumor animation than other treatments and revealed that the amalgamation of siEGFR and TMZ through pH-responsive nanocarrier was able to improve TMZ sensitivity in treating GBM.

The survival time of glioma-bearing mice varied with different treatments. The control group (PBS) exhibited the minimum survival time of 25.5 days. In contrast, the longest survival time appeared in the group treated with Tf-PEG-PAE(SS)/TMZ@siEGFR, with the median survival time being 64.5 days, longer than that of the free TMZ (36.5 days), TMZ@siEGFR (43.5 days), and PEG-PAE(SS)/TMZ@siEGFR (48 days) treatments (Figure 10E). The change trends in the survival rate were also reflected in body weights. Similarly, the weight of mice with the treatment of Tf-PEG-PAE(SS)/TMZ@siEGFR decreased slowly, while that of other groups decreased rapidly (Figure 10F). These results indicated that the simultaneous delivery of TMZ and siEGFR through the tumor microenvironment responsive nanocarrier could achieve a synergistic antiglioma effect.

**FIGURE 11:**
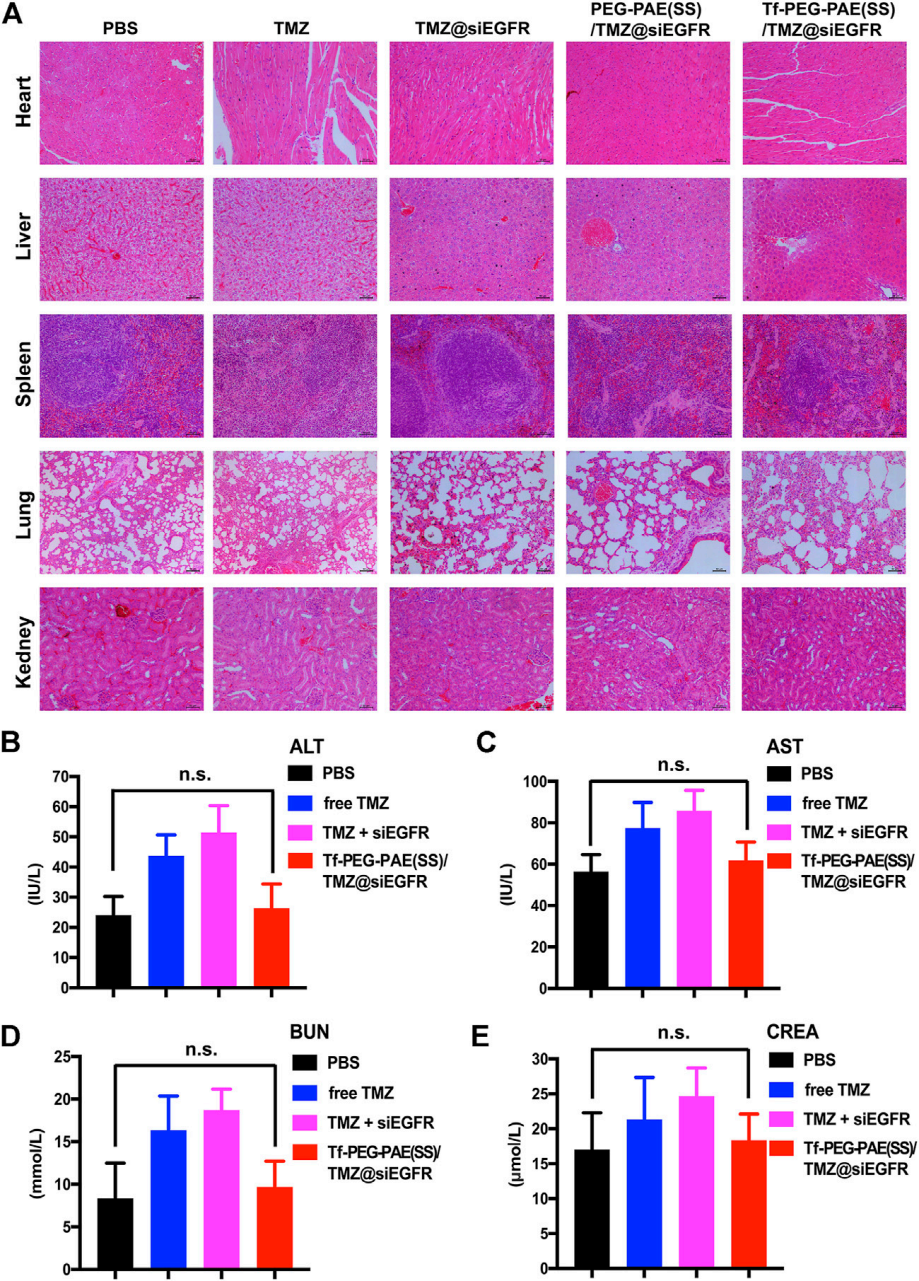
Toxicity study of Tf-PEG-PAE(SS)/TMZ@siEGFR. **(A)** H&E staining of the heart, liver, spleen, lung, and kidney. **(B–E)** Biochemical indication levels of ALT, AST, BUN, and CREA of mice after different treatments. (Mean ± SD, n = 3); n.s. indicates no statistical significance.

The *in vivo* toxicity of Tf-PEG-PAE(SS)/TMZ@siEGFR was evaluated by major organs (the heart, liver, spleen, lung, and kidney) through hematoxylin and eosin (H&E) staining (Figure 11A). H&E-stained images showed no obvious tissue damage in the free TMZ, TMZ@siEGFR, PEG-PAE(SS)/TMZ@siEGFR or Tf-PEG-PAE(SS)/TMZ@siEGFR group. compared with the PBS group. Moreover, the biochemical indication levels of ALT, AST, BUN, and CREA in the serum were also detected. The results indicated these biochemical indications had no conspicuous change after Tf-PEG-PAE(SS)/TMZ@siEGFR treatment (Figures 11B–E). The *in vivo* experimental results demonstrated that the pH-responsive co-loading nanoparticles Tf-PEG-PAE(SS)/TMZ@siEGFR had a good biocompatibility and could further realize the clinical transformation application.

## Conclusion

In summary, a pH-sensitive nanoparticle based on polymer PEG-PAE (SS), with TMZ encapsulated in the core and siEGFR complexed by the cationic layer, was studied. Both *in vitro* and *in vivo* experiments demonstrated extremely efficient co-delivery of the two therapeutic agents into U87 glioma cells. Tf-PEG-PAE(SS)/TMZ@siEGFR nanoparticles were kept stable under neutral pH conditions but efficiently disassembled for rapid drug release inside the acidic environment of cancer cells. Therefore, the antiapoptotic EGFR gene was silenced, and meanwhile, the TMZ concentration was heightened, enhancing tumor cell apoptosis. Furthermore, in the animal study, due to the efficient targeted transport and pH-responsive release, the combinative therapy of TMZ and siEGFR restrained the hyperplasia of GBM and prolonged median survival of mice dramatically. Consequently, the co-loading of TMZ and siEGFR through Tf-PEG-PAE(SS) could display a striking anticancer efficacy and few side effects in glioma treatment, providing a promising co-delivery system for the clinical application.

## Data Availability

The datasets presented in this study can be found in online repositories. The names of the repository/repositories and accession number(s) can be found in the article/Supplementary Material.
